# The influence of partial union on the mechanical strength of scaphoid fractures: a finite element study

**DOI:** 10.1177/17531934231157565

**Published:** 2023-02-22

**Authors:** Esin Rothenfluh, Sambhav Jain, Roman Guggenberger, William R. Taylor, Seyyed Hamed Hosseini Nasab

**Affiliations:** 1 Department of Plastic and Hand Surgery, University Hospital Zurich, Zurich, Switzerland; 2 Laboratory for Movement Biomechanics, Swiss Federal Institute of Technology (ETH) Zurich, Zurich, Switzerland; 3 Institute of Diagnostic and Interventional Radiology, University Hospital Zurich, Zurich, Switzerland

**Keywords:** Scaphoid, scaphoid fracture, finite element modelling, biomechanics, fracture union

## Abstract

Assessment of scaphoid fracture union on computed tomography scans is not currently standardized. We investigated the extent of scaphoid waist fracture union required to withstand physiological loads in a finite element model, based on a high-resolution CT scan of a cadaveric forearm. For simulations, the scaphoid waist was partially fused at the radial and ulnar sides. A physiological load of 100 N was transmitted to the scaphoid and the minimal amount of union to maintain biomechanical stability was recorded. The orientation of the fracture plane was varied to analyse the effect on biomechanical stability. The results indicate that the scaphoid is more prone to re-fracture when healing occurs on the ulnar side, where at least 60% union is required. Union occurring from the radial side can withstand loads with as little as 25% union. In fractures more parallel to the radial axis, the scaphoid seems less resistant on the radial side, as at least 50% union is required. A quantitative CT scan analysis with the proposed cut-off values and a consistently applied clinical examination will guide the clinician as to whether mid-waist scaphoid fractures can be considered as truly united.

## Introduction

Most scaphoid fractures occur at the waist or mid-third of the bone (Garala et al., 2016) and are considered to be displaced when there are steps or gaps of at least 1 mm (Dias and Singh, 2011). There has been an increase in the use of screw fixation as 10% to 15% of minimally or undisplaced fractures do not heal (Clay et al., 1991). However the use of primary treatment by surgical fixation of these fractures was not supported by the level I evidence provided in the scaphoid waist internal fixation for fractures trial (SWIFFT) study of surgery versus cast immobilization for adults with bicortical fracture of the scaphoid waist (the SWIFFT study) (Dias et al., 2020): there was no significant difference in the patient-rated wrist evaluation score between the surgical and cast immobilization groups at 52 weeks. This supports the initial use of non-operative treatment for undisplaced mid-waist fractures of the scaphoid but any suspected nonunion should be detected early and fixed surgically (Dias et al., 2020). The criteria for union on a computed tomography scan are currently not standardized and it is often difficult to establish whether the fracture is healed or not at different stages of treatment. This is mainly because the appearance of trabecular bridging can often be observed only in a specific proportion of the fracture plane (Singh et al., 2005). Quantifying this proportion would be helpful for the study and treatment of scaphoid fractures, and lead to more rational clinical decision-making for both surgical and non-operative treatment.

The aim in this study was to quantify, in a finite element model of the wrist, how much of the whole width of the scaphoid waist should be bridged with trabecular bone to represent biomechanical stability under physiological loads. In a second step, we varied the angulation of the fracture plane to investigate any effect on the cut-off values for adequate fracture consolidation.

## Methods

In agreement with the ethical regulations of the cantonal ethics committee in Zurich (Ethical Approval No.: 2020-02842, Date 07.01.2021), cadaveric human forearms were obtained from donors who had voluntarily donated their bodies to the Anatomy Institute of the University of Zurich. A suitable Thiel fixated sample was chosen by the principal investigator (ER), based on normal bony architecture of the wrist with absent osteoarthritic changes, no implants and regular intercarpal intervals and angles.

### Geometry

**Figure 1.**
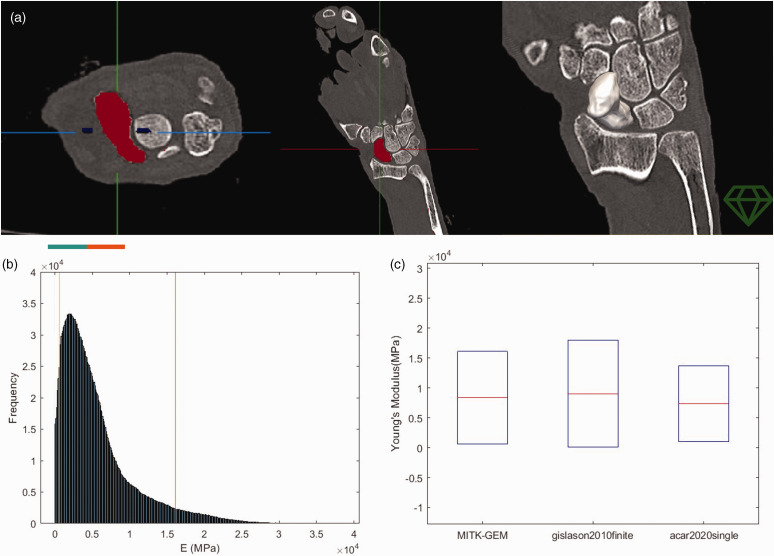
(a) Segmentation of the scaphoid bone into fore- and background elements, based on CT scan data (image courtesy of MITK-GEM). (b) A histogram of the elastic modulus of the mesh was plotted and compared against published values for validation and (c) Ninety per cent of the elastic modulus values in the mesh were within the ranges previously reported.

High-resolution computed tomography scans with the wrist in neutral position were taken with the following settings: 100 kVp tube voltage, 12 mA tube current, 0.8 revolution time, 512 × 512 pixels image matrix, 0.625 mm slice thickness with 2 mm spacing. Voxel sizes were 160/512, equal to 0.3125 mm in-plane and 0.625 mm through-plane. The geometry of the scaphoid was extracted from the CT data, which was segmented using a graph cut technique within the publicly available MITK-GEM software (Medical Interaction Toolkit Workbench, http://araex.github.io/mitk-gem-site/) (Figure 1(a)). A surface and a volume mesh were generated with tetrahedral elements (up to 0.5 mm in size), and the CT data converted into a computer-aided design (CAD) model. The MITK-GEM Software was used to map the bone material properties onto the CAD geometry. Here, we used the Hounsfield unit of each voxel to calculate the apparent bone density (*ρ_app_* in milligrams of hydroxyapatite per cubic centimetre; mgHA/cm^3^), based on an empirical relationship. The mean value of Young’s modulus, *E*, in megapascals (MPa) was then calculated from the apparent bone density of each element according to the formula: *E = *6.850(*ρ_app_*)^1.49^ (Oftadeh et al., 2015). The mineral bone density was translated into an ash density by using: *ρ_app_* = *ρ_ash_*_/_0.6 (Ganghoffer and Goda, 2018; Helgason et al., 2008), which led to the ultimate tensile strength (in MPa) according to: $\sigma_{u}\;=\;{\text{117}}_{\rho_{\mathrm{ash}}}^{{\;}^{\text{1}.\text{93}}}$ . The mean ultimate strength of the scaphoid was found to be 60.5 MPa.

### Validation

For confirmation of the material mapping, a histogram of the elastic modulus of the mesh was generated and compared with published data (Figure 1(b)), showing that approximately 90% of the elements had an elastic modulus within the ranges previously reported (Figure 1(c)). The bone mesh together with the material property data were imported into ANSYS® (Academic Research Mechanical, Release 18.1; https://www.ansys.com/academic) for static structural analysis. A test simulation with an intact scaphoid was carried out and loading conditions copied from previous publications, showing comparable results including a realistic stress distribution and displacement (Acar et al., 2018; Luria et al., 2010).

### Loading and boundary conditions

**Figure 2.**
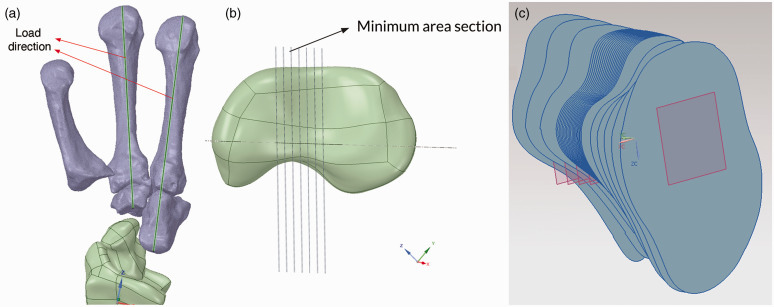
(a) To simulate physiological loading conditions, the load vector was applied at the location of ligamentous attachments on the scaphoid, aligned through the index and middle metacarpal bones, based on previously published experimental data. (b, c) Different planar cross-sections of the scaphoid were considered with a minimum spatial resolution of 0.1 mm to find the plane at greatest risk of fracture (Image courtesy of ANSYS®, Inc.).

For the main scaphoid analysis, boundary conditions were based on an area of fixed support between the proximal pole of the scaphoid and the radial scaphoid fossa. The exact size and location of the fixed area was calculated with the help of previously published data from pressure film analysis in cadavers during load transfer experiments (Viegas et al., 1989). The mean value of the radioscaphoid contact area was 40.3 mm^2^ (SD 13.1) in a neutral position based on published analysis of MRI data (Sasagawa et al., 2009). To simulate the physiological loading conditions within the wrist, the determination of the load vector direction and magnitude was also based on previously published experimental data (Viegas et al., 1989). The load was then applied at the location of ligamentous attachments on the scaphoid according to Varga et al. (2016) (Figure 2(a)). Based on earlier publications, 200 N was used as a reasonable value for the force passing through the whole wrist under physiological loading conditions (Ezquerro et al., 2007; Luria et al., 2010; Shepherd and Johnstone, 2002; Youm and Flatt, 1984). The force–transmission ratio through the radioscaphoid contact surface has been estimated to be between 44% and 55% of the total transmitted force, resulting in about 100 N at the radioscaphoid articulation (Ezquerro et al., 2007; Manal et al., 2002; Schuind et al., 1995). This represents the threshold force that must be withstood to ensure biomechanical stability of the scaphoid. According to rigid body model analysis, the normal resultant force in the radioscaphoid joint under physiological conditions in the adult population is 88.2 N (SD 13.3) (Schuind et al., 1995). In order to analyse the stress behaviour of the scaphoid, the magnitude of force was varied from 21.1 to 300 N. The first value, 21.1 N, was chosen on the basis of studies by Varga et al. (2016) in which muscle forces during gripping of a 0.5 kg object were simulated and cartilage contact forces acting on the scaphoid from neighbouring bones were analysed. Viegas et al. (1989) varied the applied forces in their experiments using the values 49, 103, 205 and 409 N. Similarly, we started with 21 N and increased the force in steps, including 21, 100, 212 and 300 N (Luria et al. 2010).

### Finite element model analysis

**Figure 3.**
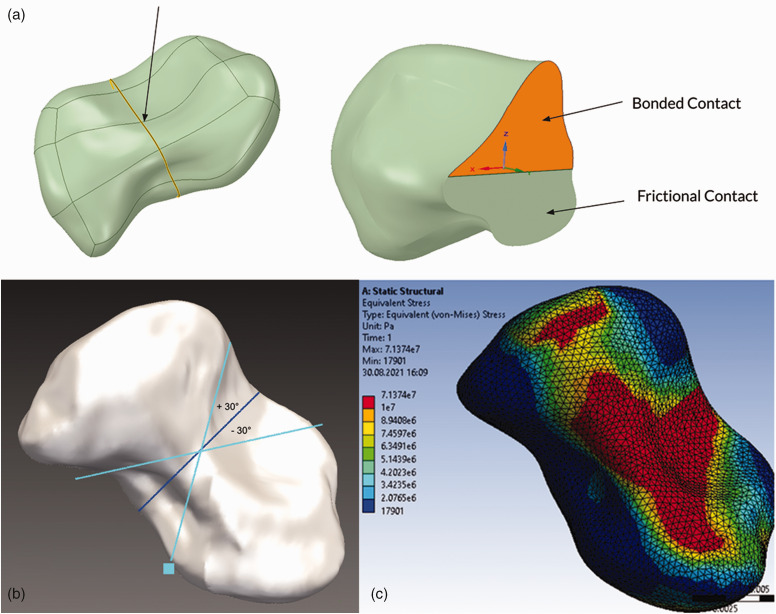
(a) The fracture plane was partially fused, and the non-fused area was simulated with a frictional contact. (b) Variation of spatial orientation of the fracture plane by 30° in two directions, representing a proximal oblique or distal oblique fracture. (c) In all simulations under loading, peak stress values were located on the radiopalmar aspect of the scaphoid waist in the model (image courtesy of ANSYS®, Inc.).

Finite element analysis was carried out using the ANSYS® modelling suite. Different planar cross-sections of the scaphoid were considered, with a minimum spatial resolution of 0.1 mm (Figure 2(b) and (c)). The variation of the cross-sectional area along the longitudinal axis was plotted and the smallest cross-sectional area in the CAD geometry chosen to divide the scaphoid bone and represent a mid-waist fracture. The fracture plane was progressively fused in a stepwise manner, representing different stages of fracture healing. The non-fused proportion of the cross-section was defined with a frictional contact (Figure 3(a)), with a coefficient of friction of 0.4, based on the finite element modelling published by Varga et al. (2016). The fracture plane was decreased in 25% steps from the radial or ulnar side, respectively, based on the grading system published by Singh et al. (2005), and force values of 21, 100, 212 and 300 N were applied as outlined above. To approximate the cut-off value more accurately, the plane of the most frequent waist fracture was decreased in 5% steps from the ulnar and radial sides for waist fractures orthogonal to the long axis.

Based on the ultimate strength of the scaphoid (60.5 MPa), the load at the onset of bone failure was determined for various healing stages. Subsequently, spatial orientation of the fracture plane was varied by an angle of 30° in two directions, representing a fracture almost parallel to the radial axis and one nearly orthogonal to the radial axis (Herbert B1 pattern) (Figure 3(b)). In all the simulated cases, the maximum amount of stress and deformation occurring in the fracture plane were recorded.

Biomechanical stability of the scaphoid fracture was assumed to be maintained as long as the ultimate strength of the scaphoid (60.5 MPa) was not exceeded. The minimum amount of union that was required to guarantee biomechanical stability under physiological loading conditions was then recorded as the cut-off value for failure.

## Results

**Figure 4.**
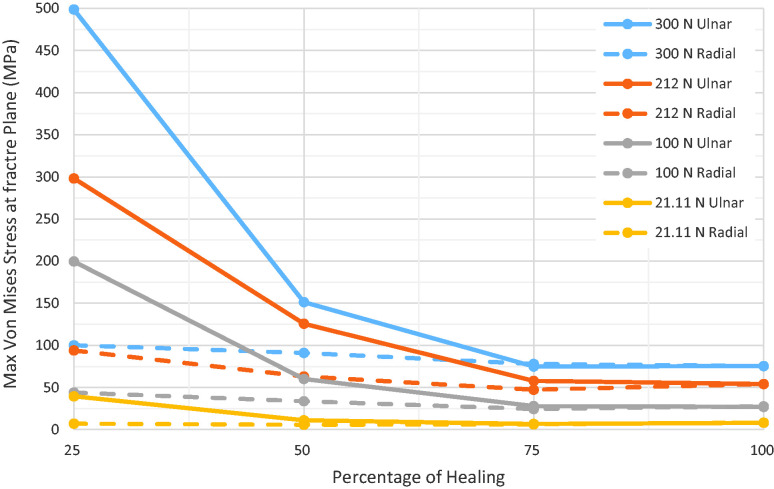
For every stage of union, the magnitude of force was varied between 21 N and 300 N, in order to plot maximum stress (MPa) within the scaphoid as a function of fracture union (%). The red line represents the ultimate sustainable stress of the scaphoid. The intersection with an individual curve represents the threshold of fracture union to withstand the load constituted by the curve. The grey curves show the results for physiological loading (100 N). The shading marks the area under the curves, which may be considered as biomechanically stable.

In all simulations, peak stress values were located on the ulnopalmar aspect of the scaphoid waist (Figure 3(c)). The stage of union was plotted against maximum stress values for each applied force, where 100 N represents the physiological loading threshold that must be withstood without exceeding the ultimate strength of the scaphoid (Figure 4).

**Figure 5.**
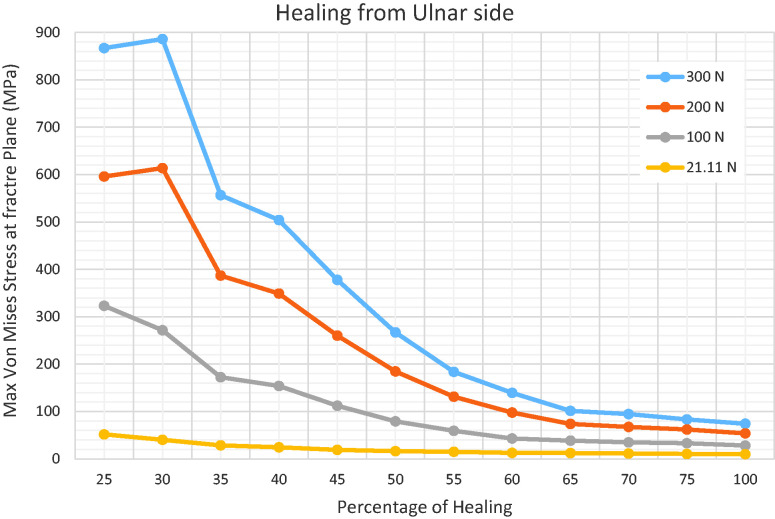
Fine analysis with 5% incremental steps of bone healing shows that the precise cut-off healed area to avoid failure was 60% union on the ulnar side of the scaphoid.

**Figure 6.**
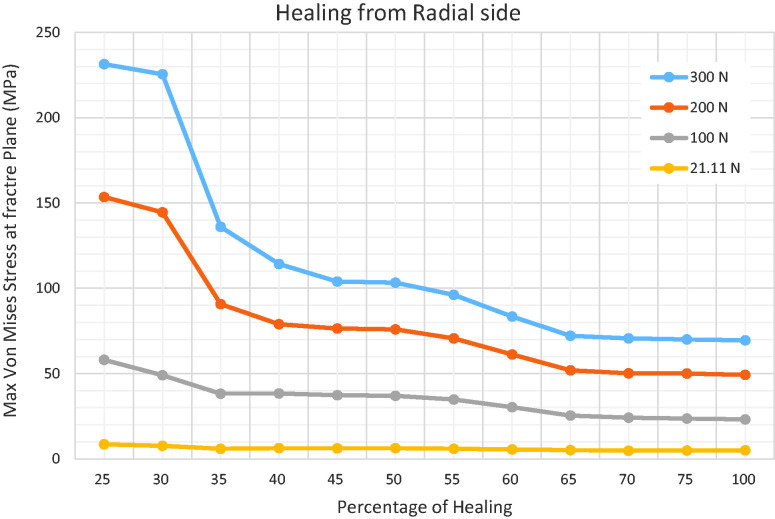
The precise cut-off healed area to avoid failure was 25% on the radial side of the scaphoid.

The analysis showed that 75% of the fracture plane (or more) must be healed on the ulnar side to withstand physiological loads. When there was 25% or 50% union, the maximum stress values were too high (249 MPa) or on the threshold of failing (60 MPa), making a re-fracture of the scaphoid very likely. In the subsequent, more detailed, analysis, 5% incremental steps of bone healing were studied, starting on the most ulnar side of the waist fracture. Based on this, the precise cut-off value for failure was in fact 60% of the union area (Figure 5). The same approach was applied on the radial aspect of the scaphoid. Here, the cut-off value was found to be 25% for the amount of union that represents biomechanical stability on the radial side of the scaphoid (Figure 6). When healing is more progressed on the radial side, higher loads than 100 N are required for material failure. Such loads are not expected to occur during common activities of daily living (Table 1).

**Table 1.** Summary of simulation results with stress values for each combination of fracture union (%) and load.

Healing	21.1 N	100 N	200 N	300 N
Intact	5	25	51	**72**
25% ulnar	41	**200–249**	**300–520**	**336–761**
50% ulnar	10	**60**	**100–157**	**100–236**
75% ulnar	5.2	26	55	**71–75**
25% radial	7.8	45	**95**	**80–120**
50% radial	5.7	34	**55–70**	**90**
75% radial	4.2	23	40–54	**76**

Bold = failure; bold and underline = borderline stress values.

In the case of physiological loading (100 N) at least 50% of fracture union is required on the ulnar side. The decrease in stress between different stages of union seems big, necessitating a more detailed analysis shown in Figure 5.

**Figure 7.**
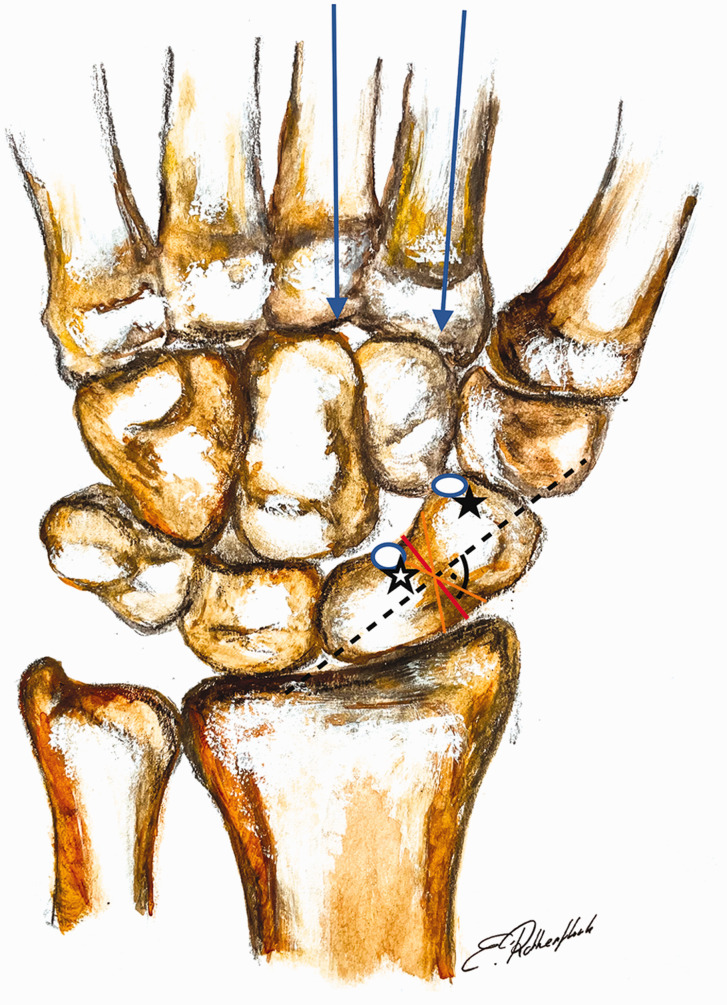
This image demonstrates the relationship between the load vectors and spatial orientation of the fracture plane. The more parallel the fracture plane to the radial axis (black star), the higher the stress values due to shear. A more horizontal fracture lessens the maximum stress values (white star).

Subsequent analyses focused on the stress distribution in an oblique fracture plane. If the plane was rotated by 30°, simulating a fracture approaching the axis of the radius (Figure 3(b)), 50% of the fracture had to be bridged by trabecular bone on the radial side to ensure biomechanical stability. If healing occurred on the ulnar side, no differences were observed in the cut-off value for withstanding physiological loads compared with results at 0° of obliquity. If the fracture plane was rotated away from the radial axis, the stress values under physiological activity did not exceed the ultimate strength when there was healing on the radial side with union of just 25%. On the ulnar side, only 50% healing was required to ensure biomechanical stability. It can therefore be concluded that when the orientation of the fracture plane is closer to the axis of the radius, a larger proportion of healed bone is required to avoid refracture, especially on the radial aspect. Here, 50% rather than 25% healing is required to provide sufficient biomechanical strength. In contrast, fractures that are orthogonal to the radial axis experience lower levels of stress (Figure 7).

## Discussion

There are no standardized criteria for the determination of union in scaphoid fractures on CT scans. It is therefore not precisely defined when a fracture may be regarded as a nonunion and at what time point immobilization is no longer required. We aimed to assess the biomechanical strength of partial fracture union in a finite element model, which may suggest quantitative criteria for union. The results of our study suggest that the scaphoid is more prone to re-fracture under physiological loading conditions when healing occurs from the ulnar side in a waist fracture orthogonal to the long axis. In that case, 60% union is required to withstand physiological daily loads. Union occurring from the radial side can withstand physiological loads with as little as 25% union. However, in fractures parallel to the radial axis, the bone seems to be less resistant on the radial side, requiring 50% to be healed. Our findings correlate the extent of healing to mechanical stability, which helps to determine when to remove a cast and advise on return to work.

CT scanning has been used not only to estimate union, but also to determine how much of the fracture gap is bridged by healing bone (Amirfeyz et al., 2011; Singh et al., 2005). The percentage of union in CT scans has also been used in previous studies, particularly when dealing with waist fractures (Grewal et al., 2013). However, the interpretation of the degree of union based on CT images remains somewhat descriptive if there are no quantitative reference standards. Singh et al. (2005) studied the incidence of partial union on CT scans at 12 to 18 weeks and investigated whether partial union may still progress to union, depending on the percentage of trabecular bone bridging. In their study, they categorized union along the cross section of the scaphoid into four groups: 0%–24%, 25%–49%, 50%–74% and 75%–100%. The authors concluded that partial union greater than 25% of the scaphoid is a common occurrence and progresses to full union in most cases. Grewal and colleagues (2013) also made use of computed tomography to assess factors that might have a predictive value for union of non-operatively treated scaphoid waist fractures. An arbitrary cut-off value of 50% of union on the CT scan was chosen and the fracture was classified as not united if 50% union was not achieved. This value has been discussed in a study by Amirfeyz et al. (2011), in which fractures with bone resorption affecting 50% or less of the cross-section of the fracture on a week 4 CT scan were associated with a significantly higher rate of union compared with those fractures with more than 50% bone resorption. These findings concur with our suggested cut-off values since biomechanical stability of the scaphoid will correspond to a higher union rate.

Computational modelling involves simplification of a biological system and therefore has its limitations, which also apply to our model. For instance, we did not include ligaments as this would have increased processing time significantly and our loading results using an intact scaphoid correlated well enough with results from other studies (Acar et al., 2018). The force application in our model did not consider the agonist and antagonist activities of flexor and extensor muscles of the forearm (force couples), which have been included in other models (Ezquerro et al., 2007; Luria et al., 2010). Although the wrist is not thought of as a weightbearing joint like the knee, hip or ankle, it takes considerable loads in various activities and these might exceed the loads we applied. Nevertheless, taking 100 N as the transmitted load to the scaphoid represented physiological loading and is in keeping with previously published data (Ezquerro et al., 2007; Manal et al., 2002; Shepherd and Johnstone, 2002). In our model, the healed part of the fracture was represented by bone and not callus. Consequently, there was no consideration of time-dependant variation of tensile mechanical properties (Christel et al., 1981; Han et al., 2016). The bone mineral density (BMD) has been quantified before in a study of conservatively treated scaphoid fractures using high-resolution peripheral quantitative computed tomography scans (HRpQCT) at different time points; this revealed a significant decrease in the first 6 weeks of bone healing (Bevers et al., 2021). In addition, they estimated the load to failure with micro-finite element analysis, showing decreased bone strength in this first healing phase. These findings suggest that mechanical strength is a time-dependent process and must be considered as a dynamic variable. Their study, however, only included a small sample of nine patients and the simulations were based on pure mechanical testing without representing physiological conditions. The load to failure was estimated with the criterion of Pistoia et al. (2002), a method to estimate load to failure using whole bone micro finite element (mFE) analysis. However, local strains can be inaccurate with this method, when calculated at specific points within the trabecular bone tissue, for instance at a fracture site (Ladd and Kinney, 1998). Therefore, a direct comparison of our results against those reported by Bevers and co-workers (2021) is not appropriate. The question about when immobilization can be stopped is, in practice, also more relevant at or beyond the generally accepted minimum 6 weeks of conservative treatment. Therefore, a decrease of BMD or estimated load to failure in the first healing phase is unlikely to have a major impact.

Other previous finite element models of the scaphoid were established with homogenous material properties, which is not reliable owing to the non-uniform distribution of material properties of the scaphoid (Acar et al., 2018; Varga et al., 2016). In our study, we were able to map the material property composition of the CT scan onto the model, which would be expected to deliver more realistic values.

Another limitation of our study might be the way in which bone healing was simulated. Bone healing might occur in a diffuse and simultaneous manner across the fracture and not segmentally, namely on either the ulnar or radial side, as was simulated in our study. In our experience, however, scaphoid fractures most often heal faster on one side of the waist, similar to our modelling.

The results of this study offer cut-off values for the stability of scaphoid waist fractures based on a quantitative finite element analysis with different degrees of consolidation under physiological loading. As a rule of thumb, we would suggest that about two-thirds of the fracture plane must be healed on the ulnar or about one-third on the radial side to ensure biomechanical stability in waist fractures that are orthogonal to the long axis of the scaphoid. This suggests that the radial part of the scaphoid waist is more crucial in the healing process, as less trabecular bridging is required to withstand physiological loads. We also determined how spatial variation of the fracture plane contributes to stability; we found that in fractures parallel to the radial axis, a larger portion must be healed on the radial aspect, making this type of fracture more prone to instability. These findings should help in treating scaphoid fractures by avoiding prolonged immobilization.
